# Effect of Sacubitril/Valsartan on Reducing the Risk of Arrhythmia: A Systematic Review and Meta-Analysis of Randomized Controlled Trials

**DOI:** 10.3389/fcvm.2022.890481

**Published:** 2022-07-01

**Authors:** Ruxin Wang, Haowen Ye, Li Ma, Jinjing Wei, Ying Wang, Xiaofang Zhang, Lihong Wang

**Affiliations:** ^1^Department of Endocrinology and Metabolism, The First Affiliated Hospital of Jinan University, Guangzhou, China; ^2^Department of Functional Examination, Gansu Provincial Maternal and Child Health Hospital, Lanzhou, China; ^3^Clinical Experimental Center, The First Affiliated Hospital of Jinan University, Guangzhou, China

**Keywords:** sacubitril/valsartan, arrhythmia, atrial arrhythmia, ventricular arrhythmia, cardiac arrest, ACEI, ARB

## Abstract

**Background and Objective:**

Relevant data of PARADIGM-HF reveals sacubitril/valsartan (SV) therapy led to a greater reduction in the risks of arrhythmia, and sudden cardiac death than angiotensin converting enzyme inhibitor (ACEI)/angiotensin receptor inhibitor (ARB) therapy in HFrEF, however, inconsistent results were reported in subsequent studies. Here, we conduct a meta-analysis of related randomized controlled trials (RCTs) to evaluate the protective effect of SV on reducing the risk of arrhythmias.

**Methods and Results:**

RCTs focused on the difference in therapeutic outcomes between SV and ACEI/ARB were searched from PUBMED, EMBASE, ClinicalTrials.gov, and Cochrane Library. The results were extracted from each individual study, expressed as binary risk, 95% confidence interval (CI) and relative risk (RR). Sixteen RCTs including 22, 563 patients met the study criteria. Compared with ACEI/ARB therapy, SV therapy did significantly reduce in the risks of severe arrhythmias among patients with heart failure with reduced ejection fraction (HFrEF) (RR 0.83, 95% CI 0.73–0.95, *p* = 0.006), ventricular tachycardia (VT) among patients with HFrEF (RR 0.69, 95% CI 0.51–0.92, *p* = 0.01), cardiac arrest among patients with heart failure (HF) (RR 0.52, 95% CI 0.37–0.73, *p* = 0.0002), cardiac arrest among patients with HFrEF (RR 0.49, 95% CI 0.32–0.76, *p* = 0.001), cardiac arrest or ventricular fibrillation (VF) among patients with HF (RR 0.63, 95% CI 0.48–0.83, *p* = 0.001), and cardiac arrest or VF among patients with HFrEF (RR 0.65, 95% CI 0.47–0.89, *p* = 0.008), but reduced the risks of arrhythmias (RR 0.87, 95% CI 0.74–1.01, *p* = 0.07), atrial arrhythmias (RR 0.98, 95% CI 0.83–1.16, *p* = 0.85), and atrial fibrillation (RR 0.98, 95% CI 0.82–1.17, *p* = 0.82) among all patients with no significant between-group difference. The merged result was robust after sensitivity analysis, and there was no publication bias.

**Conclusion:**

Our meta-analysis provides evidence that, compared with ACEI/ARB, SV can additionally reduce the risks of most arrhythmias, just the significant differences are revealed in reducing the risks of VT, severe arrhythmias, and cardiac arrest in patients with HFrEF. Besides, the positive effect of SV on VF according to statistical result of combining VF with cardiac arrest in patients with HFrEF is credibility.

## Introduction

Arrhythmia is one of the most common diseases in cardiovascular field, which has complex relationships with multiple diseases (1), especially heart failure (HF). Destruction of normal periodicity and regularity of the electrical activity in heart has been recognized as the mechanism of arrhythmia. Atrial fibrillation (AF) and ventricular arrhythmia (VA) are common and serious in patients with HF, which can cause various complications and ultimately lead to disability or death, such as thrombus caused by AF, sudden cardiac death (SCD) caused by sustained ventricular tachycardia (VT) or ventricular fibrillation (VF), etc. (2). The risk of AF has increased 3-fold in the world over the past 50 years based on the results of Framingham Heart Study (3), and the global prevalence of AF was about 46.3 million in 2016 according to the estimate of WHO (4). AF is the most common persistent arrhythmia with an average prevalence of 25% in HF (5), which can increase the risks of stroke and death (6). Sustained VA is a serious complication of HF, as it accounts for 75–80% of SCD, while about 30–50% of cardiovascular death in patients with HF was attributed to SCD (7). Arrhythmia is a challenging problem especially under the circumstance of HF, as their mutual interaction could further aggravating the state of an illness. Therefore, received widespread attention in terms of prevention, diagnosis, and treatment of arrhythmia. At present, a part of arrhythmias can be cured by new technologies with the wider application of interventional and surgical therapy (8), nevertheless, drug therapy is still currently the most important mean of the prevention and treatment of arrhythmias due to economic, scope of application of new technologies, etc.

The data of PARADIGM-HF prompted that sacubitril/valsartan (SV) therapy led to a greater reduction in the risk of SCD and significant survival benefit of SCD observed from the Kaplan-Meier curves than enalapril therapy among patients with heart failure with reduced ejection fraction (HFrEF), indicating SV may directly reduce the risk of SCD (9). Since then, the effect of SV on arrhythmia has attracted widespread concern. The effect of SV in arrhythmia is uncertain although it has been fully affirmed in HF and hypertension (10, 11). The researches of Russo et al. (12), Tsai et al. (13), and Curtain et al. (14) revealed that SV could improve ventricular remodeling while reducing the risk of VA compared with angiotensin converting enzyme inhibitor (ACEI)/angiotensin receptor inhibitor (ARB), meanwhile, it could improve atrial remodeling and atrial arrhythmia by the researches of Suo et al. (15) and Li et al. (16). Nevertheless, different results on arrhythmias were reported by El-Battrawy et al. (17), Martens et al. (18), Solomon et al. (19), Velazquez et al. (20), and McMurray et al. (10), etc.

Thus far, few specifically studies about the effect of SV on arrhythmia have been investigated. The purpose of this meta-analysis is to provide some new approaches for the treatment of arrhythmia by analyzing the risks of arrhythmias in randomized controlled trials (RCTs) for SV.

## Materials and Methods

### Search Strategy

We searched the Cochrane Library, Embase, PubMed and ClinicalTrials.gov, the current four major medical databases, which contain the vast majority of medical research literatures, as of February 21, 2022, and it was re-run twice on March 3, 2022. The search conditions include: sacubitril valsartan, sacubitril/valsartan, sacubitril, entresto, LCZ696, AHU377, angiotensin receptor neprilysin inhibitor, neprilysin inhibitor, sacubitril valsartan sodium hydrate, sacubitril valsartan drug combination. There was no protocol for expected registration, but the search terms, inclusion criteria, and data collection forms were pre-specified in the analysis plan and remain unchanged during data collection and analysis. The publication date and language restrictions were not applied, and reference lists of related articles were also used to supplement search terms. The study included only RCT.

### Study Selection

The trials included in this study need to meet the following conditions: (1) The trial is an RCT. (2) The control group is intervened with ACEI/ARB, the experimental group is intervened with SV. (3) All studies must have data on adverse events endpoint or adverse reaction of arrhythmias.

### Data Extraction

The two researchers independently extracted data from RCTs that met the criteria and Cochrane reviewer’s handbook. In the event of discrepancies, all authors discussed the results. The research data was retrieved from the original published manuscript or the results in ClinicalTrials.gov. Extracted the following data from each trial: 1. Name of the trial, author, registration number; 2. year of publication; 3. number of people enrolled; 4. characteristics of participants at baseline, including arrhythmia, age, gender, etc.; 5. drug in control group; 6. study duration; 7. main outcome.

### Quality Assessment

Two researchers separately assessed the risk of bias for each qualified trial by the Cochrane Collaboration’s tool and compiled a bias risk table as described in Cochrane Handbook (21). The quality items used to evaluate each study were as follows: sequence generation of allocation, allocation concealment, blinding of participants, blinding of outcome assessment, incomplete outcome data, selective outcome reporting, and other biases.

### Outcome Measures

The number of arrhythmias listed in results of trials, including atrial arrhythmia, AF, VA, etc.

### Statistical Analyses

Data of arrhythmias was analyzed by Review Manager 5.4, sensitivity analysis and publication bias detection by Stata 17.0, and *I*^2^ was used to assess heterogeneity. *I*^2^ ≥ 50% or the corresponding *P*-value (*p* < 0.05) was considered to have obvious heterogeneity results and then we used a random model. *I*^2^ < 50% and the corresponding *P*-value (*p* > 0.05) was considered to have no obvious heterogeneity results and then we used a fixed model (22). The results were extracted from each trial, expressed as binary risk, 95% confidence interval (CI), and relative risk (RR). The Mantel-Haenszel method and Z test were used to determine the overall results to determine the significance of RR. The heterogeneity was assessed with the *I*^2^ test, *P* < 0.05 was considered statistically significant. All results are in line with the declarations of PRISMA and Meta-analysis (23).

### Publication Bias and Sensitivity Analysis

Publication bias was judged using funnel plot, Begg and Berlin (24), and Egger’s test (25), *P* < 0.05 was considered statistically significant.

## Results

### Description of Selected Studies

**FIGURE 1:**
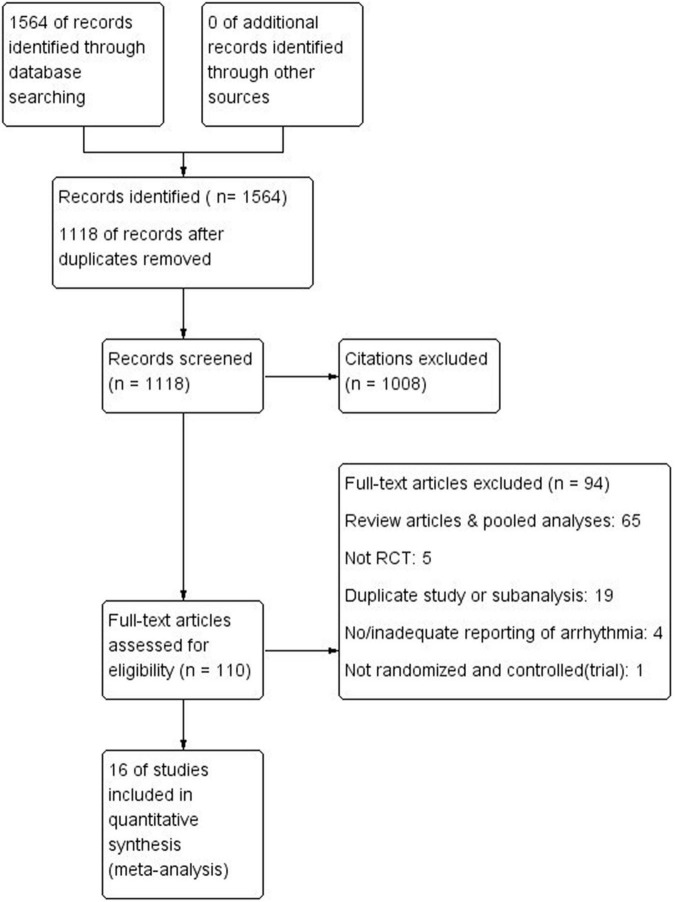
Study flow diagram.

A total of 1, 564 possible articles or studies were initially identified, and 446 possible articles were left after filtered repeated research by endnote. The remaining articles were judged by the two researchers according to criteria, final 16 RCTs were included for analysis. The flow chart is shown in Figure 1.

### Study Characteristics

The detailed characteristics of 16 studies (10, 19, 20, 26–38) are shown in Table 1. Most of the data are come from ClinicalTrials.gov, 14 trials are multi-center and 12 trials are aimed at patients with HF. The total number of subjects is 22, 563 and the follow-up ranged from 8 weeks to 33.9 months. All are RCTs.

**TABLE 1:** Characteristics of included RCTs.

Trial	Number	Follow-up	Patient	Inclusion criteria	Age (years)	Male (%)	Control	Dosage	Baseline arrhythmia (%)	Arrhythmia outcome (%)	Main outcome
EVALUATE-HF (29)	464	12 weeks	Hypertension with HFrEF	Age ≥ 50 years, hypertension, CHF and EF ≤ 40%, NYHA I-III	67.8 ± 9.8 vs. 66.7 ± 8.5	355 (77)	Enalapril	200 mg bid vs. 10 mg bid		Arrhythmia: 5 (2) vs. 0 (0) AA: 2 (0.9) vs. 0 (0) VA: 2 (0.9) vs. 0 (0) cardiac arrest: 0 (0) vs. 0 (0)	Treatment of HFrEF with SV, compared with enalapril, did not significantly reduce central aortic stiffness
NCT01785472 (30)	1,438	8 weeks	Hypertension	Mean sitting SBP ≥ 140 to < 180 mm Hg	(57.5 ± 10.17, 58.1 ± 9.71) vs. 57.4 ± 10.14	756 (53)	Olmesartan	200/400 mg qd vs. 20 mg qd		Arrhythmia: 0 (0) vs. 1 (0.2) AA: 0 (0) vs. 1 (0.2)	Treatment with SV once daily is effective and provided superior BP reduction than olmesartan in Asian patients with mild-to-moderate hypertension
NCT01615198 (26)	588	14 weeks	Hypertension	Mean sitting SBP ≥ 140 to < 180 mm Hg, aged ≥ 65 years	70.5 ± 4.67 vs. 70.9 ± 4.67	294 (50)	Olmesartan	Starting dose: 100 mg qd vs. 10 mg qd maximum dose: 400 mg qd vs. 40 mg qd		Arrhythmia: 2 (0.7) vs. 0 (0) AA: 1 (0.3) vs. 0 (0)	SV is more effective than olmesartan in reducing BP in elderly Asian patients with systolic hypertension
NCT01599104 (31)	1,161	8 weeks	Hypertension	Japanese patients aged ≥ 20 years with mild to moderate systolic hypertension	(57.9 ± 10.9, 58.7 ± 10.5) vs. 59.6 ± 10.5	818 (70.5)	Olmesartan	200–400 mg qd vs. 20 mg qd		Arrhythmia: 0 (0) vs. 1 (0.3)	Treatment with SV was effective and provided superior BP reduction, with a higher proportion of patients achieving target BP goals than treatment with olmesartan in Japanese patients with mild to moderate essential hypertension
ACTIVITY-HF (32)	201	12 weeks	HFrEF	Aged ≥ 18years with CHF [NYHA III and EF ≤ 40%] and an objectively reduced exercise capacity (peak VO_2_ ≤ 18 mL/min/kg%)	66.1 ± 10.8 vs. 67.6 ± 10.0	163 (81)	Enalapril	200 mg bid vs. 10 mg bid		Arrhythmia: 5 (0.5) vs. 4 (0.4) AA:2 (0.2) vs. 0 (0) VA: 2 (0.2) vs. 0 (0)	In patients with HFrEF, short-term treatment with SV for 12 weeks did not result in significant benefits on peak VO2 when compared with enalapril
Wang Q (33)	149	3 months	HFpEF	Persistent AF (> 7 days or < 7 days but requiring electrical or pharmacological cardioversion) and HF symptoms	58.9 ± 12.75 vs. 62.7 ± 10.91	94 (68.12)	Valsartan	100 mg bid vs. 80 mg bid	All patients had AF	Arrhythmia: 15 (22) vs. 29 (42) AA: 15 (22) vs. 29 (42)	SV can decrease AF recurrence after catheter ablation in patients with persistent AF at the 1-year follow-up
Wang H (34)	137	24 weeks	HFrEF	Acute anterior STEMI, 18 years ≤ age < 75 years, LVEF < 45% and SBP ≥ 100 mmHg	59.13 ± 7.15 vs. 60.56 ± 7.62	106 (77)	Enalapril	Starting dose: 50/100 mg bid vs. 2.5/5 mg bid		Arrhythmia: 6 (9) vs. 9 (13)	SV attenuated LV remodeling and dysfunction and was safe and effective in LV systolic dysfunction patients post-acute anterior wall myocardial infarction
PARAMOUNT (28)	301	3 months	HFpEF	NYHA II-III HFpEF, EF > 45%	70.9 ± 9.4 vs. 71.2 ± 8.9	152 (57)	Valsartan	200 mg bid vs. 160 mg bid	History of AF: 60 (40) vs. 65 (43) AF at screening: 40 (27) vs. 45 (30)	Arrhythmia: 5 (3) vs. 16 (11) AA:3 (2) vs. 9 (6)	SV has better effect on reducing BNP, improving LA reverse remodeling and NYHA compared with the valsartan in patients with HFpEF
PIONEER-HF (20)	881	8 weeks	HFrEF	Hemodynamic stabilization after ADHF and EF ≤ 40%	61 (51, 71) vs. 63 (54, 72)	635 (72.1)	Enalapril	200 mg bid vs. 10 mg bid	AF: 147 (33.4) vs. 165 (37.4)	Arrhythmia: 13 (3) vs. 20 (5) AA: 6 (1) vs. 4 (1) VA: 5 (1) vs. 8 (2) cardiac arrest: 0 (0) vs. 4 (1)	Among patients with HFrEF who were hospitalized for ADHF, the initiation of SV therapy led to a greater reduction in the NT-proBNP concentration than enalapril therapy
PRIME (39)	118	12 months	HFrEF	NYHA II-III, EF > 25% and < 50%, significant functional MR lasting > 6 months	64.7 ± 10.2 vs. 60.5 ± 11.8	72 (61)	Valsartan	200 mg bid vs. 160 mg bid	AF: 15 (25.9) vs. 16 (26.7)	Arrhythmia: 0 (0) vs. 1 (2) VA:0 (0) vs. 1 (2)	Among patients with secondary functional MR, SV reduced MR to a greater extent than did valsartan
OUTSTEP-HF (36)	621	12 weeks	HFrEF	NYHA II and LVEF ≤ 40%	66.89 ± 10.74	487 (79)	Enalapril	200 mg bid vs. 10 mg bid	AF: 147 (47.57) vs. 122 (39.35) SVT: 16 (5.18) vs. 9 (2.90)	Arrhythmia: 18 (6) vs. 19 (6) AA: 8 (3) vs. 6 (2) VA: 5 (1.6) vs. 2 (0.6) cardiac arrest: 0 (0) vs. 2 (0.6)	There was no significant benefit of SV either 6MWT or in daytime physical activity measured by actigraphy compared with enalapril
PARALLEL-HF (37)	223	33.9 months	HFrEF	NYHA II-IV and EF ≤ 35%	69.0 ± 9.7 vs. 66.7 ± 10.9	192 (86)	Enalapril	200 mg bid vs. 10 mg bid	AFL: 36 (32.4) vs. 40 (35.7)	Arrhythmia: 11 (10) vs. 12 (11) AA: 4 (4) vs. 4 (4) VA: 7 (6) vs. 8 (7)	In Japanese patients with HFrEF, there was no difference in reduction in the risk of cardiovascular death or HF hospitalization between SV and enalapril
PARALLAX (38)	2 566	24 weeks	HFpEF	NYHA II-IV, EF > 40%, LV hypertrophy or left atrial enlargement with NT-proBNP↑	73 ± 8.4 vs. 72 ± 8.6	1,265 (49)	Enalapril /valsartan	200 mg bid vs. 10 mg bid vs. 160 mg	AF or AFL: 699 (54.6) vs. 692 (53.9)	Arrhythmia: 10 (1) vs. 15 (1) AA: 10 (0.9) vs. 15 (1.3)	Among patients with HFpEF, SV treatment compared with standard renin angiotensin system inhibitor treatment or placebo resulted in a significantly greater decrease in NT-proBNP levels at 12 weeks but did not significantly improve 6MWT at 24 weeks
PARADIGM-HF (10)	8 442	27 months	HFrEF	NYHA II-IV,EF ≤ 40%	63.8 ± 11.5 vs. 63.8 ± 11.3	6 567 (78)	Enalapril	200 mg bid vs. 10 mg bid	AF: 1,517 (36.2) vs. 1,574 (37.4) VA:333 (4)	Arrhythmia: 504 (12) vs. 553 (13) AA: 285 (7) vs. 269 (6) VA: 99 (2) vs. 129 (3) cardiac arrest: 30 (0.7) vs. 56 (1.3)	SV was superior to enalapril in reducing the risks of death and of hospitalization for HFrEF
PARAGON-HF (19)	4 822	26 months	HFpEF	NYHA II-IV,EF ≥ 45%	72.7 ± 8.3 vs. 72.8 ± 8.5	2 317 (48)	Valsartan	200 mg bid vs. 160 mg bid	AF or AFL: 775 (32.2) vs. 777 (32.5)	Arrhythmia: 630 (26) vs. 620 (26) AA: 448 (19) vs. 409 (17) VA: 17 (0.7) vs. 10 (0.4) cardiac arrest: 17 (0.7) vs. 30 (1.2)	SV did not result in a significantly lower rate of total hospitalizations for HF and death from cardiovascular causes among patients with HFpEF
PARAMETER (27)	454	52 weeks	Hypertension	Aged ≥ 60 years with systolic hypertension	68.2 ± 5.73 vs. 67.2 ± 5.97	237 (52)	Olmesartan	200 mg bid vs. 20 mg bid		Arrhythmia: 3 (1.3) vs. 1 (0.4) AA: 2 (0.9) vs. 1 (0.4)	Demonstrated superiority of SV vs. olmesartan in reducing clinic and ambulatory central aortic and brachial pressures in elderly patients with systolic hypertension and stiff arteries

*eGFR, estimated glomerular filtration rate; SCr, serum creatinine; uACR, urine albumin:creatinine ratio; BP, blood pressure; SBP, systolic blood pressure; NT-proBNP, N-terminal pro-B type natriuretic peptide; NYHA: New York Heart Association Functional Classification; LV, left ventricle; EF, ejection fraction; HF, heart failure; HFpEF, heart failure with preserved ejection fraction; HFrEF, heart failure with reduced ejection fraction; ADHF, acute heart failure; MR, mitral regurgitation; ↑, increase; ↓, reduce; SVT, supraventricular tachycardia; AFL, atrial flutter; AF, atrial fibrillation; AA, atrial arrhythmias; 6MWT, 6-min walk distance; STEMI, ST segment elevation myocardial infarction.*

### Quality Assessment

**FIGURE 2:**
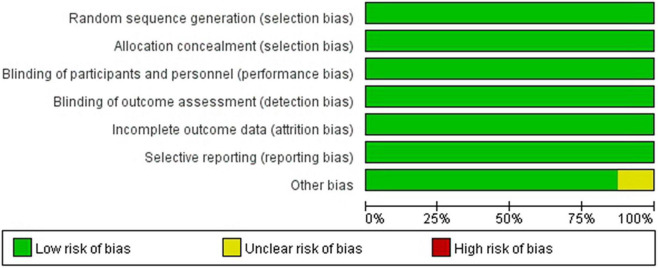
Methodological quality graph: author’s judgments about each methodological quality item presented as a percentage across all included studies.

**FIGURE 3:**
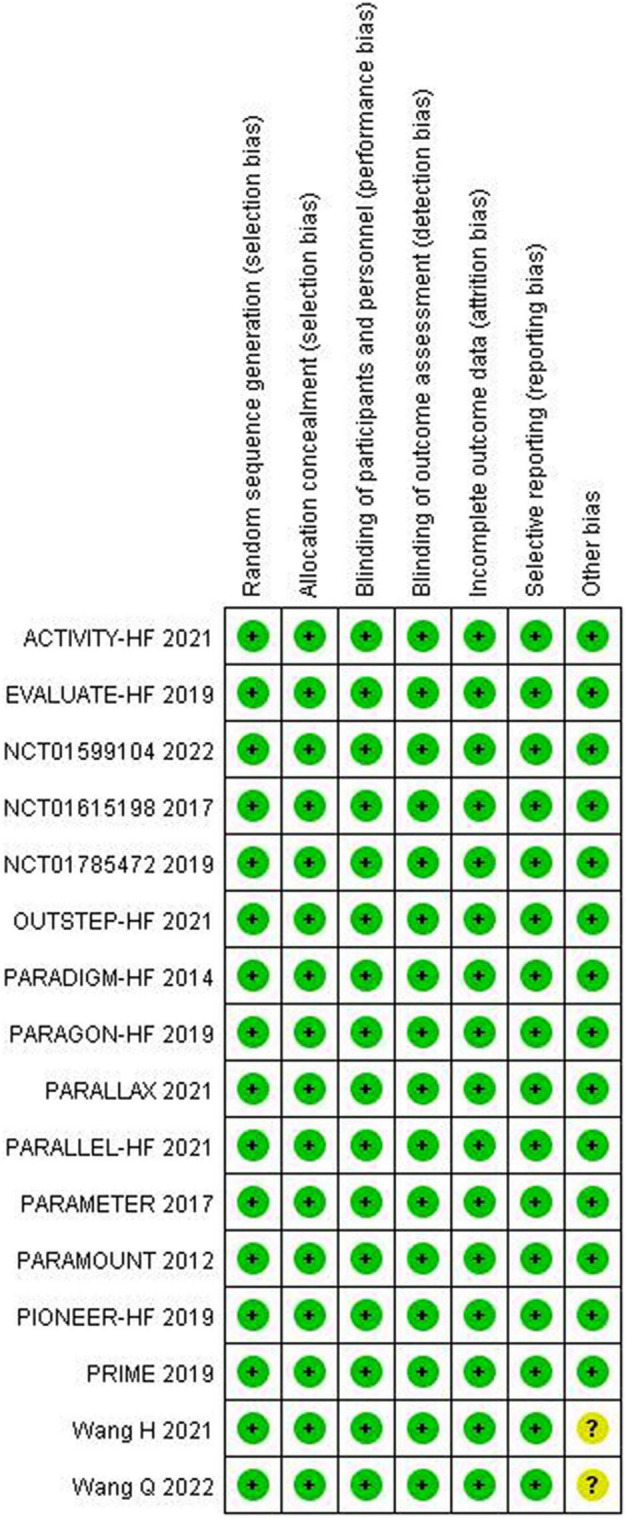
Methodological quality summary: authors’ judgments about each methodological quality.

The quality assessment for the included studies is presented in Figures 2, 3. Randomization assignment was conducted using computer-generated random numbers in a majority of the trials and prespecified outcomes were reported by all trials. Individual studies did not specifically describe the methods used to hide and allocate sequences. Overall, the included studies are of high quality.

### Clinical Outcomes Evaluation

The analysis results are summarized in Table 2.

**TABLE 2:** Results of meta-analysis.

Outcomes	RR	95% CI	*P*	No. of participants (trials)
**Arrhythmias**				
All patients	0.87	0.74–1.01	0.07	22,205 (16)
Non-HF	0.98	0.17–5.46	0.98	3,637 (4)
HF	0.87	0.74–1.01	0.07	18,568 (12)
HFrEF	0.91	0.82–1.01	0.09	11,069 (8)
HFpEF	0.69	0.36–1.31	0.26	7,361 (3)
**Severe arrhythmias**				
All patients	0.81	0.64–1.03	0.09	22,205 (16)
Non-HF	0.98	0.17–5.46	0.98	3,637 (4)
HF	0.81	0.63–1.03	0.08	18,568 (12)
HFrEF	0.83	0.73–0.95	0.006*	11,523 (9)
HFpEF	1.10	0.95–1.27	0.21	7,361 (3)
**Atrial arrhythmia**				
All patients	0.98	0.83–1.16	0.85	220,789 (13)
**AF**				
All patients	0.98	0.82–1.17	0.82	20,789 (13)
Non-HF	1.15	0.22–5.94	0.87	2,476 (3)
HF	0.97	0.79–1.18	0.73	18,313 (10)
HFrEF	1.10	0.93–1.29	0.27	10,814 (6)
HFpEF	0.69	0.41–1.16	0.16	7,499 (4)
**VAs**				
HF	0.87	0.70–1.09	0.23	15,753 (8)
HFrEF	0.82	0.64–1.03	0.09	10,932 (7)
HFpEF	1.69	0.77–3.68	0.19	4,821 (1)
**VF**				
HF	0.85	0.54–1.35	0.49	15,552 (7)
HFrEF	0.86	0.53–1.40	0.54	10,731 (6)
HFpEF	0.79	0.21–2.95	0.73	4,821 (1)
**VT**				
HF	0.76	0.58–0.99	0.04*	15,753 (8)
HFrEF	0.69	0.51–0.92	0.01*	9,716 (4)
HFpEF	2.48	0.78–7.90	0.12	4,821 (1)
**Cardiac arrest**				
HF	0.52	0.37–0.73	0.0002*	15,211 (5)
HFrEF	0.49	0.32–0.76	0.001*	10,390 (4)
HFpEF	0.56	0.31–1.02	0.06	4,821 (1)
**Cardiac arrest or VF**				
HF	0.63	0.48–0.83	0.001*	15,552 (7)
HFrEF	0.65	0.47–0.89	0.008*	10,731 (6)
HFpEF	0.60	0.35–1.02	0.06	4,821 (1)

*p < 0.05.

*HF, heart failure; HFpEF, heart failure with preserved ejection fraction; HFrEF, heart failure with reduced ejection fraction; AF, atrial fibrillation; VAs, ventricular arrhythmias; VF, ventricular fibrillation; VT, ventricular tachycardia; CI, confidence interval; RR, relative risk.*

#### The Efficacy of Sacubitril/Valsartan Compared to Angiotensin Converting Enzyme Inhibitor/Angiotensin Receptor Inhibitor on Arrhythmias

**FIGURE 4:**
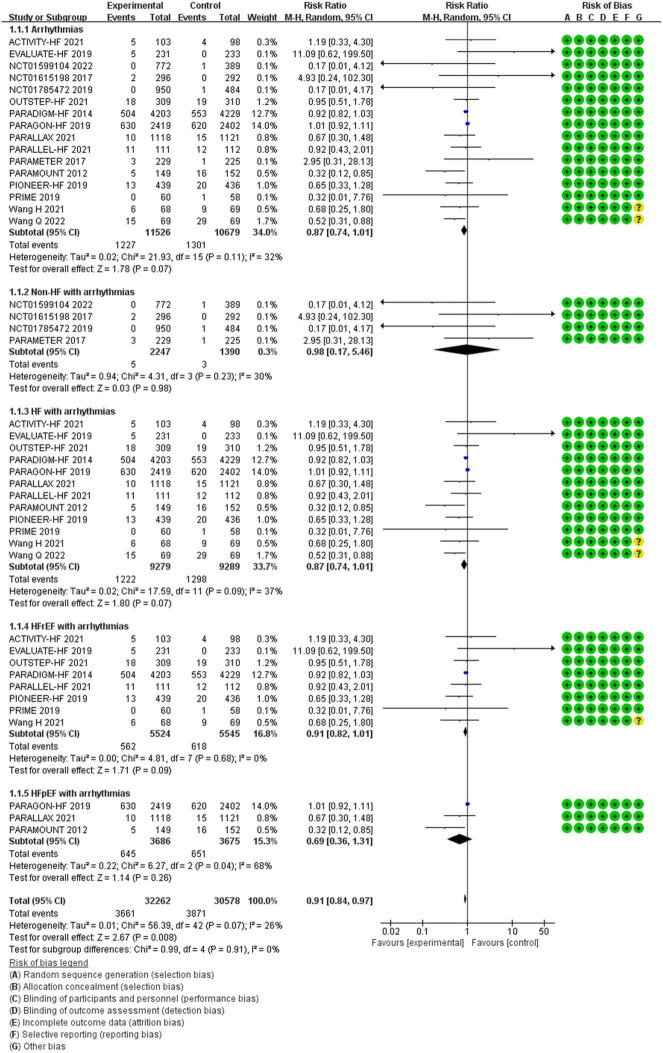
The efficacy of SV compared to ACEI/ARB on arrhythmias.

Between the two groups, the results revealed that there was no difference in reduction in the risks of arrhythmias among all patients (RR 0.87, 95% CI 0.74–1.01, *p* = 0.07), arrhythmias among patients with non-HF (RR 0.98, 95% CI 0.17–5.46, *p* = 0.98), HF (RR 0.87, 95% CI 0.74–1.01, *p* = 0.07), HFrEF (RR 0.91, 95% CI 0.82–1.01, *p* = 0.09), and HF with preserved ejection fraction (HFpEF) (RR 0.69, 95% CI 0.36–1.31, *p* = 0.26) (Figure 4).

#### The Efficacy of Sacubitril/Valsartan Compared to Angiotensin Converting Enzyme Inhibitor/Angiotensin Receptor Inhibitor on Severe Arrhythmias

**FIGURE 5:**
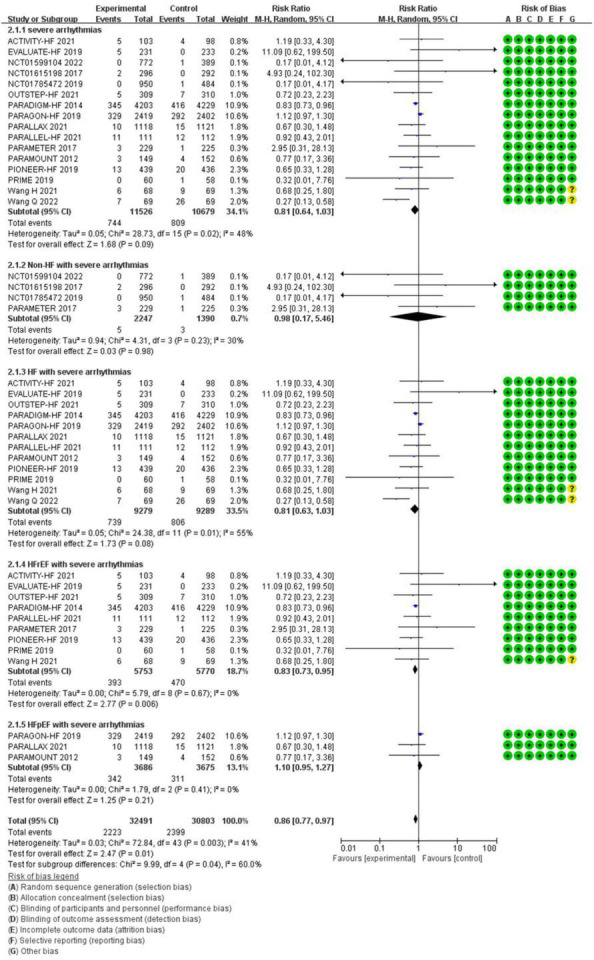
The efficacy of SV compared to ACEI/ARB on severe arrhythmias.

Compared with ACEI/ARB therapy, SV therapy did significantly reduce in the risks of severe arrhythmias among patients with HFrEF (RR 0.83, 95% CI 0.73–0.95, *p* = 0.006), but the reductions in the risks of severe arrhythmias among all patients (RR 0.81, 95% CI 0.64–1.03, *p* = 0.09), severe arrhythmias among patients with non-HF (RR 0.98, 95% CI 0.17–5.46, *p* = 0.98), HF (RR 0.81, 95% CI 0.63–1.03, *p* = 0.08), and HFpEF (RR 1.10, 95% CI 0.95–1.27, *p* = 0.21) were no significant between-group difference (Figure 5).

#### The Efficacy of Sacubitril/Valsartan Compared to Angiotensin Converting Enzyme Inhibitor/Angiotensin Receptor Inhibitor on Atrial Arrhythmias

**FIGURE 6:**
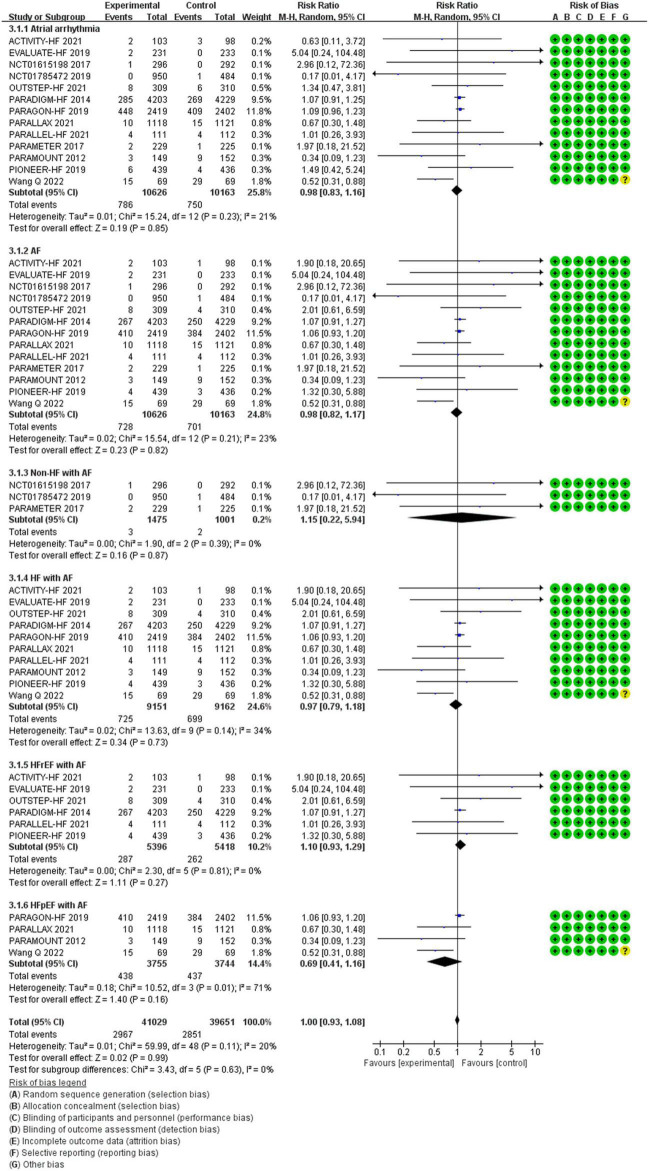
The efficacy of SV compared to ACEI/ARB on atrial arrhythmias.

Between the two groups, the results revealed that there was no difference in reduction in the risks of atrial arrhythmias among all patients (RR 0.98, 95% CI 0.83–1.16, *p* = 0.85), AF among all patients (RR 0.98, 95% CI 0.82–1.17, *p* = 0.82), AF among patients with non-HF (RR 1.15, 95% CI 0.22–5.94, *p* = 0.87), HF (RR 0.97, 95% CI 0.79–1.18, *p* = 0.73), HFrEF (RR 1.10, 95% CI 0.93–1.29, *p* = 0.27), and HFpEF (RR 0.69, 95% CI 0.41–1.16, *p* = 0.16) (Figure 6).

#### The Efficacy of Sacubitril/Valsartan Compared to Angiotensin Converting Enzyme Inhibitor/Angiotensin Receptor Inhibitor on Ventricular Arrhythmias

**FIGURE 7:**
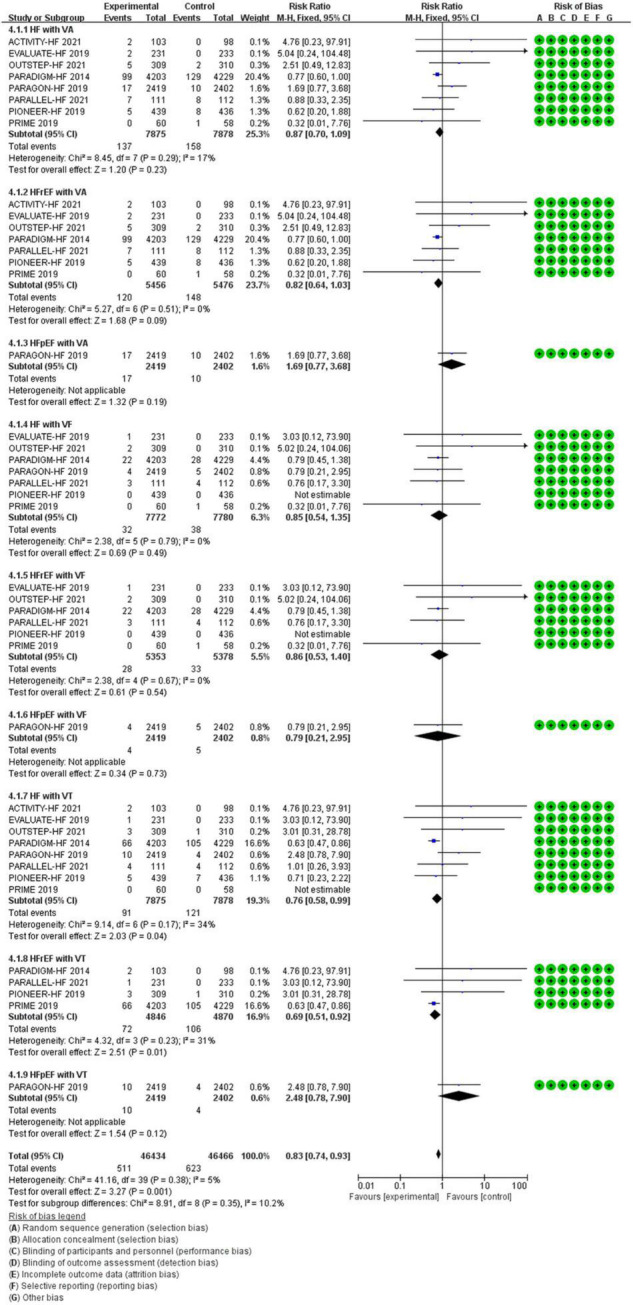
The efficacy of SV compared to ACEI/ARB on VAs.

All data of VAs were came from patients with HF. Compared with ACEI/ARB therapy, SV therapy did significantly reduce in the risks of VT among patients with HFrEF (RR 0.69, 95% CI 0.51–0.92, *p* = 0.01), but the reductions in the risks of VA among patients with HF (RR 0.87, 95% CI 0.70–1.09, *p* = 0.23), VA among patients with HFrEF (RR 0.82, 95% CI 0.64–1.03, *p* = 0.09), VA among patients with HFpEF (RR 1.69, 95% CI 0.77–3.68, *p* = 0.19), VF among patients with HF (RR 0.85, 95% CI 0.54–1.35, *p* = 0.49), VF among patients with HFrEF (RR 0.86, 95% CI 0.53–1.40, *p* = 0.54), VF among patients with HFpEF (RR 0.79, 95% CI 0.21–2.95, *p* = 0.73), and VT among patients with HFpEF (RR 2.48, 95% CI 0.78–7.90, *p* = 0.12) were no significant between-group difference (Figure 7).

#### The Efficacy of Sacubitril/Valsartan Compared to Angiotensin Converting Enzyme Inhibitor/Angiotensin Receptor Inhibitor on Cardiac Arrest or Cardiac Arrest Combined With Ventricular Fibrillation

**FIGURE 8:**
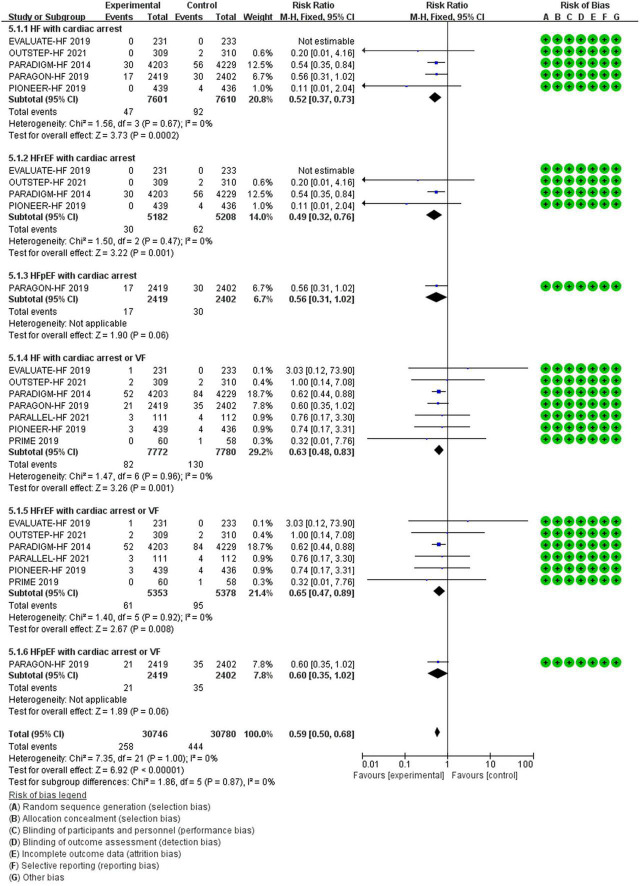
The efficacy of SV compared to ACEI/ARB on Cardiac arrest or Cardiac arrest combined VF.

Compared with ACEI/ARB therapy, SV therapy did significantly reduce in the risks of cardiac arrest among patients with HF (RR 0.52, 95% CI 0.37–0.73, *p* = 0.0002), cardiac arrest among patients with HFrEF (RR 0.49, 95% CI 0.32–0.76, *p* = 0.001), cardiac arrest or VF among patients with HF (RR 0.63, 95% CI 0.48–0.83, *p* = 0.001), and cardiac arrest or VF among patients with HFrEF (RR 0.65, 95% CI 0.47–0.89, *p* = 0.008), but the reductions in the risks of cardiac arrest among patients with HFpEF (RR 0.56, 95% CI 0.31–1.02, *p* = 0.06) and cardiac arrest or VF among patients with HFpEF (RR 0.60, 95% CI 0.35–1.02, *p* = 0.06) were no significant between-group difference (Figure 8).

### Sensitivity Analysis and Publication Bias

**FIGURE 9:**
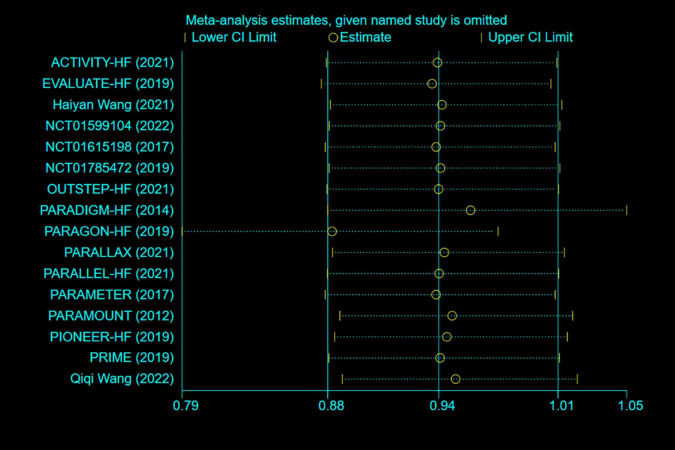
Sensitivity analysis.

**FIGURE 10:**
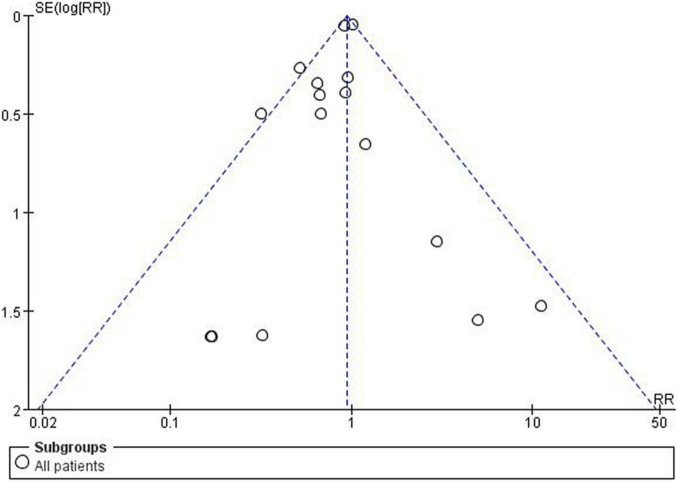
Funnel plots.

**FIGURE 11:**
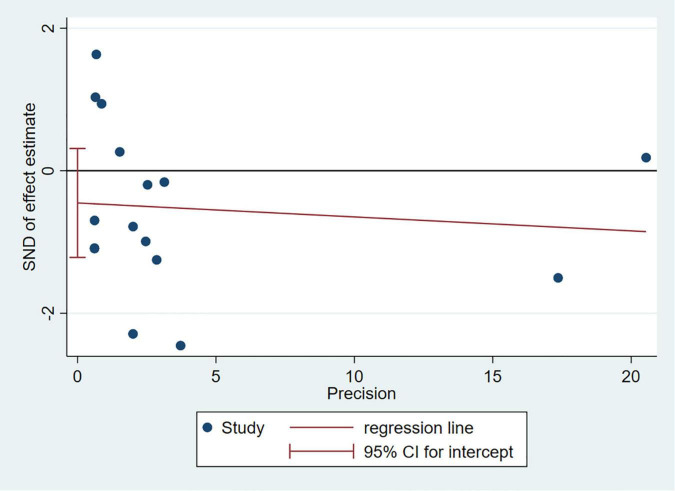
Egger’s test.

**FIGURE 12:**
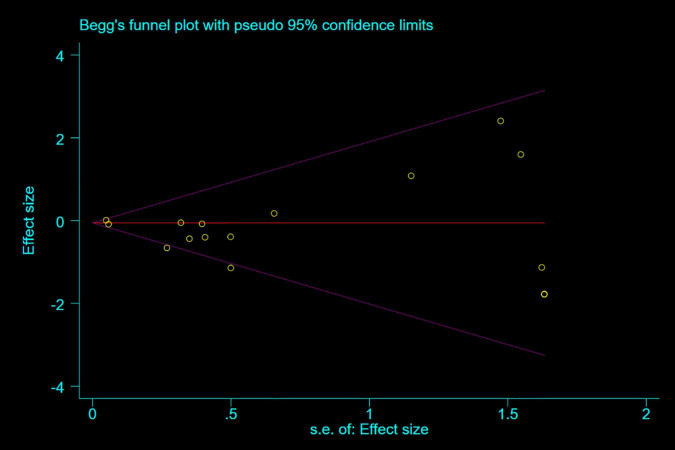
Begg’s test.

Any single study excluded would not affected the significance of our combined effect size for either outcome by sensitivity analysis (Figure 9). No obvious publication bias was found in the visual inspection of funnel plots (Figure 10), Egger’s test (Figure 11) for OS with a *p*-value of 0.225 and Begg’s Test (Figure 12) for OS with a *p*-value of 0.822 also proved it. Therefore, we could conclude that all the included studies have no obvious publication bias and the result is stable.

## Discussion

### Major Findings

Compared with ACEI/ARB therapy, SV therapy did significantly reduce in the risks of severe arrhythmias, VT and cardiac arrest among patients with HFrEF, and cardiac arrest among patients with HF by 17, 31, 51, and 48%, respectively, and the reductions in the risks of arrhythmias, severe arrhythmias, atrial arrhythmias, AF and VAs among patients with HF were 13, 19, 22, and 13%, respectively with no significant between-group difference. Although the specific relationship between the data of cardiac arrest and VF in this meta-analysis cannot be determined, considering that the main cause of cardiac arrest is VF, we combined the data for statistical analysis, and the results revealed that SV therapy did significantly reduce in reducing the risks of cardiac arrest or VF among patients with HF, HFrEF by 37 and 35%, respectively compared with ACEI/ARB therapy. Therefore, we consider that SV therapy did significantly reduce in the risks of cardiac arrest in patients with HF, mainly HFrEF, compared with ACEI/ARB therapy. Similarly, we can speculate about the superior role of SV therapy in reducing the use of implantable cardioverter-defibrillator from SV therapy led to a reduction in the risks of VT, VF, and cardiac arrest or VF compared with ACEI/ARB therapy in HFrEF. Further research is needed to confirm these speculations. Overall, the results of our meta-analysis revealed that SV therapy in specific groups are effective and provided superior arrhythmias reduction than ACEI/ARB therapy.

### Mechanisms

Cardiovascular disease mainly leads to the activation of the neuroendocrine system, including sympathetic nervous system (SNS), renin-angiotensin-aldosterone system (RAAS), and vasoactive peptides (mainly natriuretic peptides) (40). Despite the initial functional response, chronic SNS and RAAS activation increases cardiac afterload, which increasing myocardial oxygen consumption and leading to deleterious proliferative remodeling effects (41). Natriuretic peptide system (NPs) mediates the activation of cyclic guanosine monophosphate-dependent signaling pathways through corresponding receptors, resulting in vasodilation, natriuretic diuresis, and lowering of blood pressure (40). In addition, cardiac filling, preload, and ventricular remodeling are reduced by NPs and corresponding receptors through inhibiting endothelin secretion, and activation of RAAS and SNS. The effect of SV is mainly considered to enhance NPs and inhibit RAAS by inhibiting neprilysin, moreover, it has more effective dual inhibitory effect on the neuroendocrine system by combining with ARB. Furthermore, enhancing the NPs has favorable cardiovascular effects in HF and is an ideal complementary therapeutic target for RAAS and SNS blockade. Enkephalinase inhibitor (NEPI) in SV exerts natriuretic diuresis, dilates blood vessels, and reduces blood pressure by upregulating the levels of NPs, bradykinin, and adrenomedullin. Also, inhibition of sympathetic tone and RAAS can reduce aldosterone, myocardial fibrosis and hypertrophy, and ventricular remodeling. In addition to counteracting the vasoconstriction problem caused by the increased concentration of angiotensin II caused by NEPI, the combined use of valsartan can enhance the inhibition of vasoconstriction, cardiomyocyte proliferation and fibrosis, and myocardial remodeling by further inhibit SNS and decreases aldosterone levels by inhibiting sustained activation of RAAS (42, 43).

The mechanism of SV improving arrhythmia is not clear, which may be related to the treatment with SV provided superior cardiac remodeling (including structure and electrophysiology) and fibrosis (44) reduction than angiotensin inhibition, as cardiac remodeling can lead to increased susceptibility to arrhythmia and fibrosis, a well-recognized factor for malignant VA, is an important basis of cardiac remodelling (45, 46). Besides, NPs regulation has affect on myocardial electrophysiological properties and anatomical substrate, which are major factors for the development of sustained VA. The inhibition of SV on SNS and RAAS may exert a direct effect on reducing the risk of arrhythmia in HF. SNS and RAAS are activated and the secretion of neurohormone is increased in HF, lead to arrhythmia by increasing myocardial automaticity, altering conductivity and refractory periods, constricting of blood vessels to alter cardiac load, and promoting ventricular remodeling together (47, 48). In pre-clinical studies, SV improved the risk of persistent VA by reducing wall stress (49) and the activity of membrane ion channels, such as sodium channel NaV1.5 protein and potassium channel proteins, associated with VT/VF, which decrease trigger factors and perpetuating/maintaining the event of Vas (50). Moreover, demonstrated superiority of SV vs. ACEI/ARB in reducing VAs though improving potential systolic and diastolic function, calcium homeostasis and conduction delay, increased pacing threshold to induce arrhythmia, a decrease of action potential duration (APD) and the maximum slope of APD restitution by inhibiting the CaMK II pathway, and down-regulation of small-conductance Ca^2+^-activated potassium channel type 2 (44, 51, 52).

### Findings Relevant to Other Studies

The *post hoc* study of PARADIGM-HF revealed that, compared with enalapril therapy, SV therapy did significantly reduce in reducing the risk of ventricular arrhythmia and the composite arrhythmia outcome in HFrEF (14). A retrospective analysis of 1-year telemonitoring in 151 SV-treated HFrEF patients with implantable cardioverter-defibrillator/cardiac-resynchronization-therapy (18) revealed that SV treatment resulted in a reduction in mean VT/VF burden, non-sustained VT and treatments, which were associated with improved biventricular pacing and a higher degree of reverse remodeling. These results are consistent with our findings that SV therapy led to a greater reduction in the risks of VT/VF compared with ACEI/ARB therapy in HFrEF. The results of the PARADIGM-HF (9) revealed that SV had a direct effect on reducing the risk of SCD compared with enalapril, which is consistent with the findings of our study that SV therapy reduced the risk of cardiac arrest in HFrEF by 51% compared with ACEI/ARB therapy. SV therapy did not affect AF burden in the study of Martens (18), and the same is true of our study. Other than these, SV therapy could reduce the risk of severe arrhythmias by 17% in HFrEF compared with ACEI/ARB therapy. Above results are consistent with consistent with our findings, confirming the reliability of our results.

### Thoughts on Difference of Therapy Effect

Our study finds that SV therapy is superior in reducing the risk of certain arrhythmias merely in HFrEF compared with ACEI/ARB therapy, which may be related to the different therapeutic effects of SV in different EF spectra (53, 54). Although has been recommended for the treatment of HFpEF by guidelines, SV merely has a significant therapeutic advantage in morbidity among patients with HFrEF, which could be found in other large clinical trials, due to multi-factors. The pathophysiological heterogeneity within the broader clinical spectrum of HFpEF, which may represent a different progression or disease, lead to the effect of neurohormone antagonists on HFpEF is relatively weak compared with HFrEF (55). Moreover, there is a significant correlation between the degree of systolic dysfunction and frequency of arrhythmias, mainly VAs, in HFrEF, and improvement in LVEF was associated with a significant reduction in VAs and mortality (56–58). However, as normal EF of HF, HFpEF may not have a similar phenomenon. Interestingly, arrhythmias mainly existed among patients with HF in trials included, of which atrial arrhythmias are more common than VAs, and VAs mainly came from patients with HFrEF, while atrial arrhythmias mainly came from patients with HFpEF.

From the phenomena, we could speculate that HFrEF is mainly related to ventricular remodeling, while HFpEF related to atrial remodeling, and SV therapy is more advantageous in improving ventricular remodeling. Besides, differences in atrial fibrillation prevalence at baseline may have contributed to this result. Above may be the major reasons of inconsistent results in our study. Besides, the different levels of indexes of echocardiography at baseline also lead to different results of arrhythmias, such as left atrial strain and atrial volume are associated with AF (59), global longitudinal strain and mechanical dispersion are associated with VAs, etc. It is difficult to analyze the relation between indexes of echocardiography at baseline and results of arrhythmias in our study, because only EVALUATE-HF, PRIME, and Wang H provide the data of echocardiography, while few participants and inconsistent indexes are included in the trials.

### Strength and Limitations

We conducted a reasonable search of the literature and carefully screened it using strict standards, and the study included a large sample size. This is a more comprehensive analysis of the effect of SV in reducing the risk of arrhythmia, which only includes RCTs. Most of the studies in this analysis are large multicenter clinical trials, so the quality of our meta-analysis is very high. Our study confirms the advantage of SV in reducing VT, VF and cardiac arrest. However, several possible deficiencies should be noted: arrhythmias were not the main objective of these trials, the observation period of individual studies were short, most studies did not mention the occurrence of arrhythmias at baseline, only 4 trials on patients with non-HF, and the types of diseases targeted are limited. In addition, another large clinical study PARADISE-MI (ClinicalTrials.gov ID: NCT02924727) cannot be included for its data on arrhythmia has not published.

## Conclusion

This meta-analysis reveals that compared with ACEI/ARB therapy, SV therapy can reduce the risks of most arrhythmias, just the significant differences were revealed in reducing the risks of VT, severe arrhythmias, and cardiac arrest among patients with HFrEF. Besides, the positive effect of SV on VF according to statistical result of combining VF with cardiac arrest in patients with HFrEF is credibility. The result of our study provides more useful information for strengthening the clinical application of SV, especially among patients with high-risk factors for VT, VF, cardiac arrest, etc. By comparing with ACEI/ARB, we can infer that the additional antiarrhythmic effect of SV may originate from the increase of endogenous vasoactive peptides through inhibiting neprilysin. Of course, the exact mechanism and beneficiary population of SV therapy on arrhythmia need to be further clarified by further studies.

## Data Availability Statement

The original contributions presented in this study are included in the article/Supplementary Material, further inquiries can be directed to the corresponding author/s.

## Ethics Statement

All procedures strictly followed the Preferred Reporting Items for Systematic Reviews and Meta-Analyses (PRISMA). All included studies were published without ethical and informed consent dispute.

## Author Contributions

RW, HY, JW, and LM contributed to the literature database search, data collection, data extraction, data analysis, and writing of the manuscript. RW, HY, JW, and YW performed data analysis of the results. XZ and LW were conceptualized the topic. XZ and LW reviewed and revised this article. All authors contributed to the article and approved the submitted version.

## Conflict of Interest

The authors declare that the research was conducted in the absence of any commercial or financial relationships that could be construed as a potential conflict of interest.

## Publisher’s Note

All claims expressed in this article are solely those of the authors and do not necessarily represent those of their affiliated organizations, or those of the publisher, the editors and the reviewers. Any product that may be evaluated in this article, or claim that may be made by its manufacturer, is not guaranteed or endorsed by the publisher.
